# A comprehensive performance evaluation on the prediction results of existing cooperative transcription factors identification algorithms

**DOI:** 10.1186/1752-0509-8-S4-S9

**Published:** 2014-12-08

**Authors:** Fu-Jou Lai, Hong-Tsun Chang, Yueh-Min Huang, Wei-Sheng Wu

**Affiliations:** 1 Department of Engineering Science, National Cheng Kung University, Tainan, Taiwan; 2 Computational Systems Biology Lab, Department of Electrical Engineering, National Cheng Kung University, Tainan, Taiwan

## Abstract

**Background:**

Eukaryotic transcriptional regulation is known to be highly connected through the networks of cooperative transcription factors (TFs). Measuring the cooperativity of TFs is helpful for understanding the biological relevance of these TFs in regulating genes. The recent advances in computational techniques led to various predictions of cooperative TF pairs in yeast. As each algorithm integrated different data resources and was developed based on different rationales, it possessed its own merit and claimed outperforming others. However, the claim was prone to subjectivity because each algorithm compared with only a few other algorithms and only used a small set of performance indices for comparison. This motivated us to propose a series of indices to objectively evaluate the prediction performance of existing algorithms. And based on the proposed performance indices, we conducted a comprehensive performance evaluation.

**Results:**

We collected 14 sets of predicted cooperative TF pairs (PCTFPs) in yeast from 14 existing algorithms in the literature. Using the eight performance indices we adopted/proposed, the cooperativity of each PCTFP was measured and a ranking score according to the mean cooperativity of the set was given to each set of PCTFPs under evaluation for each performance index. It was seen that the ranking scores of a set of PCTFPs vary with different performance indices, implying that an algorithm used in predicting cooperative TF pairs is of strength somewhere but may be of weakness elsewhere. We finally made a comprehensive ranking for these 14 sets. The results showed that Wang J's study obtained the best performance evaluation on the prediction of cooperative TF pairs in yeast.

**Conclusions:**

In this study, we adopted/proposed eight performance indices to make a comprehensive performance evaluation on the prediction results of 14 existing cooperative TFs identification algorithms. Most importantly, these proposed indices can be easily applied to measure the performance of new algorithms developed in the future, thus expedite progress in this research field.

## Background

Transcriptional regulation is known to be highly connected through the networks of cooperative transcription factors (TFs) based on genome-wide location analysis in yeast [1,2]. Numerous studies have begun to explore how two or multiple TFs cooperate to regulate genes and what the degree of the cooperativity of these TFs is. Several types of TF-TF interactions were observed [3,4], such as (i) two TFs forming a protein complex before binding together to a TF binding site (TFBS); (ii) two TFs forming a protein complex but using only one TF to bind to a TFBS; (iii) two TFs forming a protein complex before binding to their own TFBSs; (iv) two TFs forming a protein complex to regulate another TF that binds to a TFBS and (v) two TFs compete to bind to a TFBS.

In order to make precise prediction of cooperative TF pairs, researchers developed distinct algorithms that integrated diverse genome-wide datasets such as chromatin immuno-precipitation on chip (ChIP-chip) data, gene expression data, protein-protein interaction (PPI) data, TF knockout data, position weight matrix (PWM) data, TFBS data and protein complex data. In these genome-wide datasets, ChIP-chip data were most commonly employed because they explicitly provide the binding targets of TFs. However, a TF's binding targets with low binding affinity were subject to being discarded due to stringent P-value cutoff applied to the ChIP-chip data, while they might be functionally significant with the TF [5]. Various studies manipulated the other kinds of data to either improve the identification of a TF's target genes or integrate the features found in the datasets analyzed to more accurately predict cooperative TF pairs.

On a per chronicle basis, we briefly reviewed a number of previous studies. First, Banerjee and Zhang (2003) [6] integrated ChIP-chip data and gene expression data to calculate expression correlation score and designed a model to assess the significance of the TF cooperativity based on multivariate hypergeometric distribution. Harbison et al. (2004) [7] utilized ChIP-chip data to determine co-occurring TFs whose binding sites occur more frequently in the same promoter region than random expectation. Nagamine et al. (2005) [8] employed ChIP-chip data to classify target genes of each TF pair and calculated the distances of the classified target genes based on PPI data. Tsai et al. (2005) [9] integrated ChIP-chip data and gene expression data to identify synergistic TF pairs by testing whether they are associated in the same gene more often than random expectation. Chang et al. (2006) [10] utilized ChIP-chip data and gene expression data to construct a stochastic system model to assess TF cooperativity. He et al. (2006) [11] employed ChIP-chip data and gene expression data and utilized a multivariate statistical method, ANOVA, to test whether the expression of the target genes was significantly influenced by the cooperative effect of their TFs. Wang et al. (2006) [12] integrated ChIP-chip data, gene expression data and TFBS data to develop a new framework to identify combinatorial regulation of TFs. Yu et al. (2006) [13] utilized ChIP-chip data and developed a program called Motif-PIE to identify interacting TF binding motif pairs. Elati et al. (2007) [14] utilized gene expression data and developed a data mining technique called LICORN to derive cooperative regulations. Datta and Zhao (2008) [15] employed ChIP-chip data and proposed using log-linear model to study cooperative bindings among TFs. Chuang et al. (2009) [16] integrated ChIP-chip data, gene expression data and PWM data and developed a fuzzy logic approach called ANFIS to identify potential transcriptional interactions. Wang et al. (2009) [17] employed ChIP-chip data, TFBS data, PPI data and MIPS complex catalogue data and developed a supervised learning approach to predict TF cooperativity using Bayesian networks. Lu et al. (2009) [18] integrated the functional domain annotation on protein sequences by a knowledge-based computational method to infer the cooperation between TFs. Yang et al. (2010) [19] integrated ChIP-chip data and TF knockout data to predict cooperative TF pairs by identifying the most statistically significant overlap of the target genes regulated by two TFs. Finally, Chen et al. (2012) [4] utilized ChIP-chip data and developed a method to detect the interaction of a TF pair by exploring the degree of their shared target genes.

As each algorithm integrated different data resources and was developed based on different rationales, it possessed its own merit and claimed outperforming others. However, the claim was prone to subjectivity because each algorithm compared with only a few other algorithms and only used a small set of performance indices for comparison. This motivated us to propose a series of indices to objectively evaluate the prediction performance of existing algorithms. And based on the proposed performance indices, we conducted a comprehensive performance evaluation.

## Methods

We adopted/proposed eight performance indices for comparing the performance of different algorithms in predicting cooperative TF pairs. These indices can be classified into two types: TF-based and target gene based (TG-based). The TF-based type has four performance indices which are based on the PPI partners overlap of a predicted cooperative TF pair (PCTFP), shortest path length of a PCTFP in the PPI network, the functional similarity of a PCTFP, and the overlap between a set of PCTFPs and a benchmarked set of 27 known cooperative TF pairs. The TG-based type has four performance indices which are based on the overlap of a PCTFP's target genes, the expression coherence of a PCTFP's common target genes, the functional coherence of a PCTFP's common target genes, and the PPI coherence of a PCTFP's common target genes.

### TF-based performance index 1

Yeast genes are frequently regulated through combinations of TFs [15]. As the existence of protein-protein interaction between two TFs often reflects functional similarity [3] and implies participation in the same regulation [10], the detection of cooperativity of two TFs can employ PPI data. This motivates us to propose a performance index based on the PPI partners overlap of a PCTFP using physical PPI data retrieved from BioGRID database (Release 3.2.114, July 1, 2014) [20]. Using the hypergeometric distribution [21], a score *S *is assigned to a PCTFP to represent the significance of their PPI partners overlap as follows:

(1) $$\begin{array}{c} P=\overset{\text{min}\left(N_{1},N_{2}\right)}{\underset{i=c}{\sum }}\frac{\left(\begin{array}{c} N_{1}\\ i \end{array}\right)\left(\begin{array}{c} N-N_{1}\\ N_{2}-i \end{array}\right)}{\left(\begin{array}{c} N\\ N_{2} \end{array}\right)}\\ S=-\text{log}\left(P\right) \end{array}$$

where  P is the P-value calculated using the hypergeometric distribution,  c is the number of common PPI partners of the two TFs in a PCTFP, $N_{1}$ is the number of PPI partners of the first TF, $N_{2}$ is the number of PPI partners of the second TF and N=6575 is the number of unique genes in Saccharomyces Genome Database (SGD). The greater the  S is, the more significant the cooperativity of a PCTFP is. To evaluate the performance of a set of PCTFPs from an algorithm, where each PCTFP has been given a score  S, we took the mean of these scores as the final score of this performance index.

### TF-based performance index 2

A previous study [22] observed that a biologically plausible cooperative TF pair may have a shorter path length in the physical PPI network than random expectation. This motivated us to implement a performance index based on the shortest path length of a PCTFP in the physical PPI network. The physical PPI data were retrieved from BioGRID database (Release 3.2.114, July 1, 2014) [20]. A score  S is assigned to a PCTFP as the inverse of the shortest path length of this PCTFP in the physical PPI network. The greater the  S is, the more significant the cooperativity of a PCTFP is. To evaluate the performance of a set of PCTFPs from an algorithm, where each PCTFP has been given a score  S, we took the mean of these scores as the final score of this performance index.

### TF-based performance index 3

Apart from PPI data, GO annotations were often used to computationally measure the semantic similarity of genes. Functionally similar TFs are more likely to cooperate with each other to regulate genes. This motivated us to propose a performance index based on the functional similarity of a PCTFP. The functional similarity score of a PCTFP is adopted from Yang et al.'s study [23], which used Jiang and Conrath's method [24]. The greater the functional similarity score is, the more significant the cooperativity of a PCTFP is. To evaluate the performance of a set of PCTFPs from an algorithm, where each PCTFP has been given a functional similarity score, we took the mean of these scores as the final score of this performance index.

### TF-based performance index 4

This index is adopted from Yang et al.'s study [19]. Yang et al. [19] compiled a high-quality benchmarked dataset with 27 pairs of cooperative TFs from MIPS functional complex catalogue. Then they developed a procedure based on Fisher's exact test to calculate the P-value which represents the significance of the overlap between a set of PCTFPs (from an algorithm) and the benchmarked dataset. In this study, we define a score *S *as the negative logarithm of the P-value. The greater the *S *is, the more significant the overlap between a set of PCTFPs (from an algorithm) and the benchmarked dataset is.

### TG-based performance index 1

This index (adopted from Balaji et al's study [25]) is based on significance of the overlap of a PCTFP's target genes, i.e. the significance of the associations of a PCTFP in regulating common target genes. In Balaji et al.'s study, a specific network transformation procedure was used to construct the co-regulatory network called Cnet which described the significant associations among TFs in regulating common target genes. They produced a co-regulatory coefficient dataset with 3459 TF pairs. We employed this dataset to assign a co-regulatory coefficient to each PCTFP. The greater the co-regulatory coefficient is, the more significant the cooperativity of a PCTFP is. To evaluate the performance of a set of PCTFPs from an algorithm, where each PCTFP has been given a co-regulatory coefficient, we took the mean of these coefficients as the final score of this performance index.

Various studies suggested that the transcriptional cooperativity of a TF pair can be assessed not only by the significance of the overlap of their target genes but also by the significance of the expression coherence [6], functional coherence [12] or PPI coherence [3] among their common target genes. This motivates us to propose the TG-based performance indices 2, 3 and 4 to calculate the significance of the expression coherence, functional coherence, and PPI coherence among the common target genes of a PCTFP, respectively. In this study, the common target genes of a PCTFP were retrieved from YEASTRACT database [26].

### TG-based performance index 2

This index calculates the expression coherence score (ECS) among the common target genes of a PCTFP. Let *A *be the set of all possible gene pairs formed by any two common target genes of a PCTFP. For each gene pair in *A*, its co-expression score is retrieved from the SPELL database [27]. Then the ECS is defined as the fraction of gene pairs in *A *with co-expression score higher than a threshold *T*, which was determined to be the 95^th ^percentile co-expression score value of the 39 millions of gene pairs deposited in the SPELL database. Note that 0≤ECS≤1. The greater the ECS is, the more significant the cooperativity of a PCTFP is. To evaluate the performance of a set of PCTFPs from an algorithm, where each PCTFP has been assigned an ECS, we took the mean of these ECSs as the final score of this performance index.

### TG-based performance index 3

This index calculates the functional coherence score (FCS) among the common target genes of a PCTFP. Let *A *be the set of all possible gene pairs formed by any two common target genes of a PCTFP. For each gene pair in *A*, its functional similarity score is retrieved from Yang et al.'s study [23]. Then the FCS is defined as the fraction of gene pairs in *A *with functional similarity score higher than a threshold *T*, which was determined to be the 95th percentile functional similarity score value of the 13 millions of gene pairs deposited in Yang et al.'s study [23]. Note that 0≤FCS≤1. The greater the FCS is, the more significant the cooperativity of a PCTFP is. To evaluate the performance of a set of PCTFPs from an algorithm, where each PCTFP has been assigned an FCS, we took the mean of these FCSs as the final score of this performance index.

### TG-based performance index 4

This index calculates the PPI coherence score (PCS) among the common target genes of a PCTFP. Let *A *be the set of all possible gene pairs formed by any two common target genes of a PCTFP. For each gene pair in *A*, its PPI similarity score is defined as the negative decimal logarithm of the P-value using hypergeometric distribution, which represent the significance of the overlap between the PPI partners of this gene pair. Then the PCS is defined as the fraction of gene pairs in *A *with PPI similarity score higher than a threshold *T*, which was determined to be the 95th percentile PPI similarity score value of the 14 millions of gene pairs precompiled by us using the physical PPI data retrieved from BioGRID (Release 3.2.114, July 1, 2014) [20]. Note that 0≤PCS≤1. The greater the PCS is, the more significant the cooperativity of a PCTFP is. To evaluate the performance of a set of PCTFPs from an algorithm, where each PCTFP has been assigned a PCS, we took the mean of these PCSs as the final score of this performance index.

## Results and discussion

### Categorization of 14 sets of PCTFPs under evaluation based on the data sources utilized

We adopted/proposed eight performance indices to make a comprehensive performance comparison on 14 sets of PCTFPs from 14 computational studies in the literature (See Additional file 1). Based on the data sources utilized, these studies can be categorized to eight groups, as shown in Table 1. The number of PCTFPs in different studies varies and ranges from 13 to 300.

**Table 1:** Categorization of 14 sets of PCTFPs based on data sources utilized.

Data sources utilized	Related studies	# of PCTFPs
ChIP-chip data	Harbison et al.	94
	
	Datta and Zhao	25
	
	Yu et al.	300
	
	Chen et al.	221

ChIP-chip data and gene expression data	Banerjee and Zhang	31
	
	Tsai et al.	18
	
	Chang et al.	55
	
	He et al.	30

ChIP-chip data and PPI data	Nagamine et al.	24

ChIP-chip data and TF knockout data	Yang et al.	186

ChIP-chip data, gene expression data and PWM data	Chuang et al.	13

ChIP-chip data, gene expression data and TFBS data	Wang J	14

ChIP-chip data, TFBS data, PPI data and MIPS complex catalogue data	Wang Y et al.	159

Gene expression data	Elati et al.	20

### Performance comparison using four TF-based performance indices

**Figure 1:**
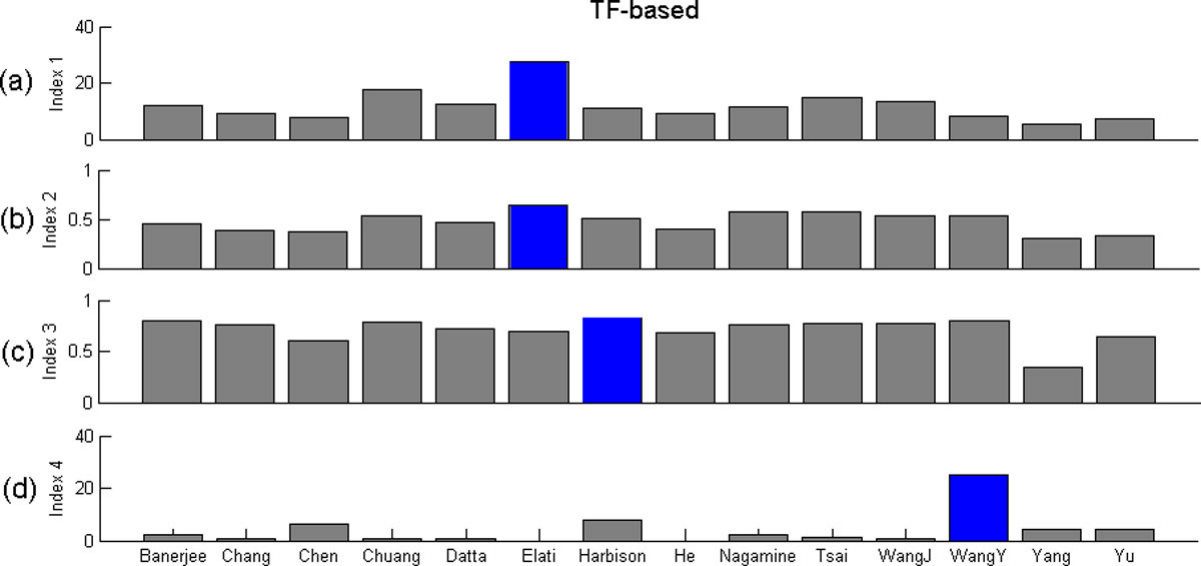
**Performance evaluation and comparison using TF-based performance indices**. (a) Index 1 is based on PPI partners overlap of a predicted cooperative TF pair (PCTFP); (b) Index 2 is based on the shortest path length of a PCTFP in the PPI network; (c) Index 3 is based on the functional similarity of a PCTFP; (d) Index 4 based on the overlap between a set of PCTFPs and a benchmarked set of 27 known cooperative TF pairs. The blue bar indicates the algorithm which outperforms the others using that performance index.

The four TF-based performance indices are developed based on the PPI partners overlap of a PCTFP, shortest path length of a PCTFP in the PPI network, the functional similarity of a PCTFP, and the overlap between a set of PCTFPs and a benchmarked set of 27 known cooperative TF pairs. Figure 1 shows the results of applying these four TF-based performance indices to each of the 14 sets of PCTFPs under evaluation. It can be seen that the result of Elati et al., Elati et al., Harbison et al. and WangY et al. outperformed the others, respectively, on index 1, 2, 3 and 4. (see Additional file 2 for details)

### Performance comparison using four TG-based performance indices

**Figure 2:**
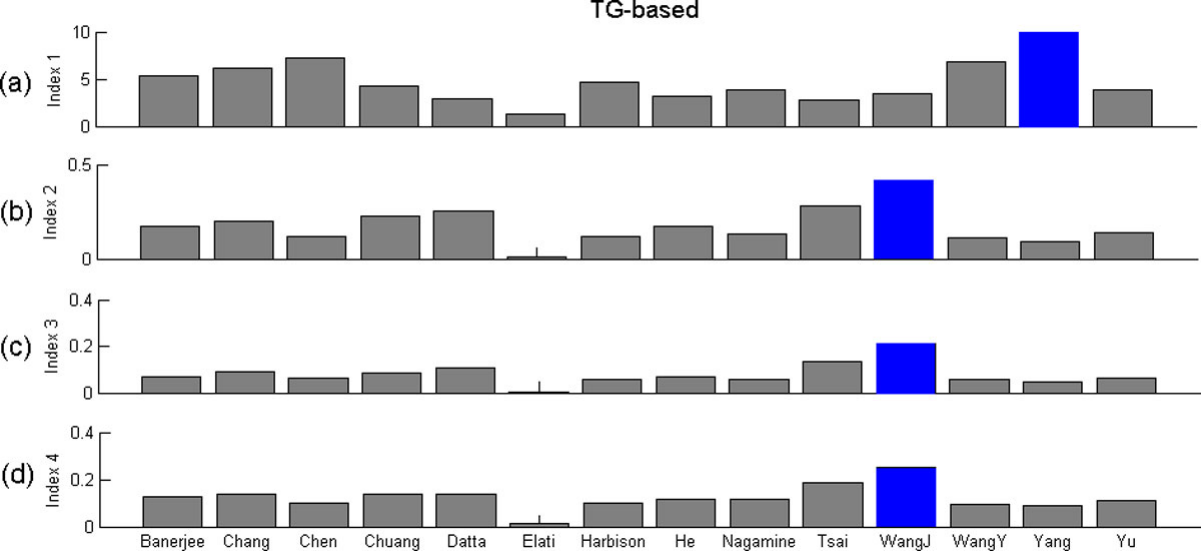
**Performance evaluation and comparison using TG-based performance indices**. (a) Index 1 is based on the overlap of a PCTFP's target genes; (b) Index 2 is based on the expression coherence of a PCTFP's common target genes; (c) Index 3 is based on the functional coherence of a PCTFP's common target genes; (d) Index 4 is based on the PPI coherence of a PCTFP's common target genes. The blue bar indicates the algorithm which outperforms the others using that performance index.

The four TG-based performance indices are developed based on the overlap of a PCTFP's target genes, the expression coherence of a PCTFP's common target genes, the functional coherence of a PCTFP's common target genes, and the PPI coherence of a PCTFP's common target genes. Figure 2 shows the results of applying these four TG-based performance indices to each of the 14 sets of PCTFPs under evaluation. It can be seen that the result of Yang et al. outperformed the others on index 1 and the result of Wang J outperformed the others on indices 2, 3 and 4 (see Additional file 2 for details).

### Comprehensive performance comparison

For each of the eight performance indices, we gave each study a ranking score, where 1 indicates the best performance while 14 the worst. Then a comprehensive ranking score of each study is defined as the sum of the ranking scores of these eight indices. The comprehensive ranking score is used to make the comprehensive performance comparison of the 14 computational studies. The smaller the comprehensive ranking score is, the better the performance of a study is. Note that the comprehensive ranking score has been used in a published paper [28], which developed several indices for comparing the performance of different microarray missing data imputation algorithms. As shown in Table 2, Wang J's study has the smallest comprehensive ranking scores, suggesting that Wang J's study obtains the best performance in predicting the cooperative TF pairs.

**Table 2:** Ranking scores given to each performance index for each study.

		Banerjee	Chang	Chen	Chuang	Datta	Elati	Harbison	He	Nagamine	Tsai	WangJ	WangY	Yang	Yu
TF-based	Idx 1	6	10	12	2	5	1	8	9	7	3	4	11	14	13
	
	Idx 2	9	11	12	6	8	1	7	10	2	3	5	4	14	13
	
	Idx 3	2	7	13	4	9	10	1	11	8	6	5	3	14	12
	
	Idx 4	6	9	3	10	12	13	2	14	7	8	11	1	4	5

TG-based	Idx 1	5	4	2	7	12	14	6	11	8	13	10	3	1	9
	
	Idx 2	7	5	11	4	3	14	10	6	9	2	1	12	13	8
	
	Idx 3	6	4	9	5	3	14	11	7	10	2	1	12	13	8
	
	Idx 4	6	5	11	3	4	14	10	8	7	2	1	12	13	9

Sum		47	55	73	41	56	81	55	76	58	39	38	58	86	77

Ranking		4	5	10	3	7	13	5	11	8	2	1	8	14	12

The comprehensive performance ranking is based on the sum of ranking scores and shown in the last row.

However, using the sum of the ranking scores is only one possible way to summarize the evaluation. Here we also provided the sum of the normalized scores as an alternative. The summarized score (SS) of algorithm *i *(i=1,2,...,14) based on the normalized score (NS) of algorithm *i *in each index *j *(j=1,2,...,8) is calculated as follows

(2) $$SS\left(i\right)=\overset{8}{\underset{j=1}{\sum }}NS_{j}\left(i\right)=\overset{8}{\underset{j=1}{\sum }}\left(\frac{OS_{j}\left(i\right)}{\text{max}\left(OS_{j}\left(1\right),OS_{j}\left(2\right),...,OS_{j}\left(14\right)\right)}\right)$$

where $OS_{j}\left(i\right)$ is the original score of algorithm *i *calculated using index *j*. Note that $0\leq NS_{j}\left(i\right)\leq 1$ and $NS_{j}\left(i\right)=1$ if and only if the algorithm *i *is the best algorithm in index *j *(i.e. it has the highest original score calculated using index *j*). The larger the summarized score is, the better the performance of a study is. As a result, we observed Wang J's study obtains the best comprehensive performance (see Table 3).

**Table 3:** Normalized scores given to each performance index for each study.

		Banerjee	Chang	Chen	Chuang	Datta	Elati	Harbison	He	Nagamine	Tsai	WangJ	WangY	Yang	Yu
TF-based	Idx 1	0.436	0.335	0.286	0.648	0.453	1	0.408	0.338	0.424	0.546	0.493	0.305	0.197	0.265
	
	Idx 2	0.719	0.596	0.582	0.834	0.737	1	0.797	0.628	0.910	0.894	0.840	0.841	0.472	0.512
	
	Idx 3	0.968	0.933	0.740	0.951	0.874	0.840	1	0.837	0.927	0.939	0.947	0.967	0.420	0.786
	
	Idx 4	0.100	0.035	0.247	0.028	0.028	0	0.315	0	0.100	0.047	0.028	1	0.177	0.164

TG-based	Idx 1	0.537	0.623	0.732	0.428	0.299	0.124	0.465	0.327	0.394	0.277	0.345	0.687	1	0.385
	
	Idx 2	0.419	0.477	0.279	0.554	0.618	0.025	0.283	0.423	0.320	0.686	1	0.275	0.226	0.335
	
	Idx 3	0.317	0.432	0.293	0.403	0.497	0.024	0.275	0.316	0.284	0.624	1	0.267	0.213	0.302
	
	Idx 4	0.505	0.546	0.407	0.561	0.554	0.048	0.411	0.465	0.473	0.747	1	0.371	0.355	0.438

Sum		4.001	3.976	3.565	4.406	4.060	3.061	3.953	3.333	3.832	4.759	5.653	4.712	3.059	3.186

Ranking		6	7	10	4	5	13	8	11	9	2	1	3	14	12

The comprehensive performance ranking is based on the sum of normalized scores and shown in the last row.

### Robustness check on using mean or median in each performance index

**Figure 3:**
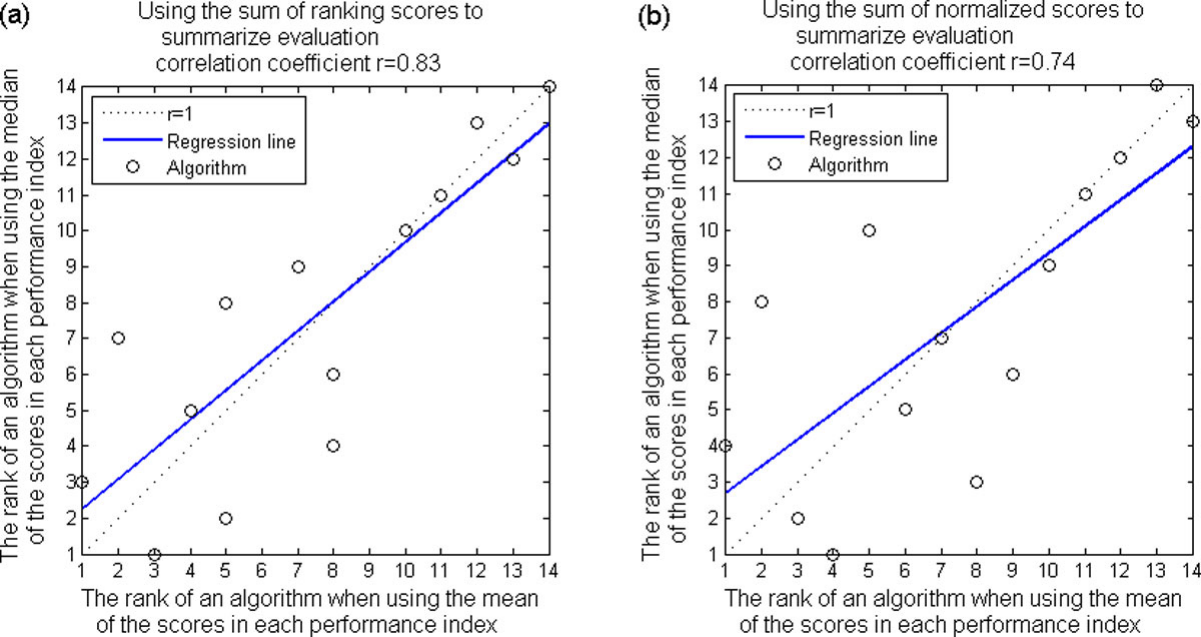
**Robustness against using mean or median of the scores in each performance index**. We compared both final ranking lists that resulted from using mean and median, and obtained (a) correlation coefficient equal to 0.83 (P-value = 2.40e-4 for testing the hypothesis of no correlation) when the sum of the ranking scores is used to summarize the evaluation and (b) correlation coefficient equal to 0.74 (P-value = 2.40e-3) when the sum of normalized scores is used to summarize the evaluation. These results suggest that the comprehensive performance comparison results are robust against using mean or median in each performance index.

In each performance index, the cooperativity of each PCTFP was measured and the mean cooperativity of the set was used as the final score of the set of PCTFPs under evaluation. We would like to see how the comprehensive performance comparison results change if we use median cooperativity of the set as the final score of the set of PCTFPs under evaluation. We compared both final ranking lists that resulted from using mean and median, and obtained correlation coefficient equal to 0.83 (P-value = 2.40e-4 for testing the hypothesis of no correlation) when the sum of ranking scores is used to summarize the evaluation. The correlation coefficient is 0.74 (P-value = 2.40e-3) when the sum of normalized scores is used to summarize the evaluation. The linear regression plots are shown in Figure 3a and Figure 3b. These results suggest that the comprehensive performance comparison results are robust against using mean or median in each performance index.

### Robustness check on using the sum of ranking scores or the sum of normalized scores to summarize evaluation

**Figure 4:**
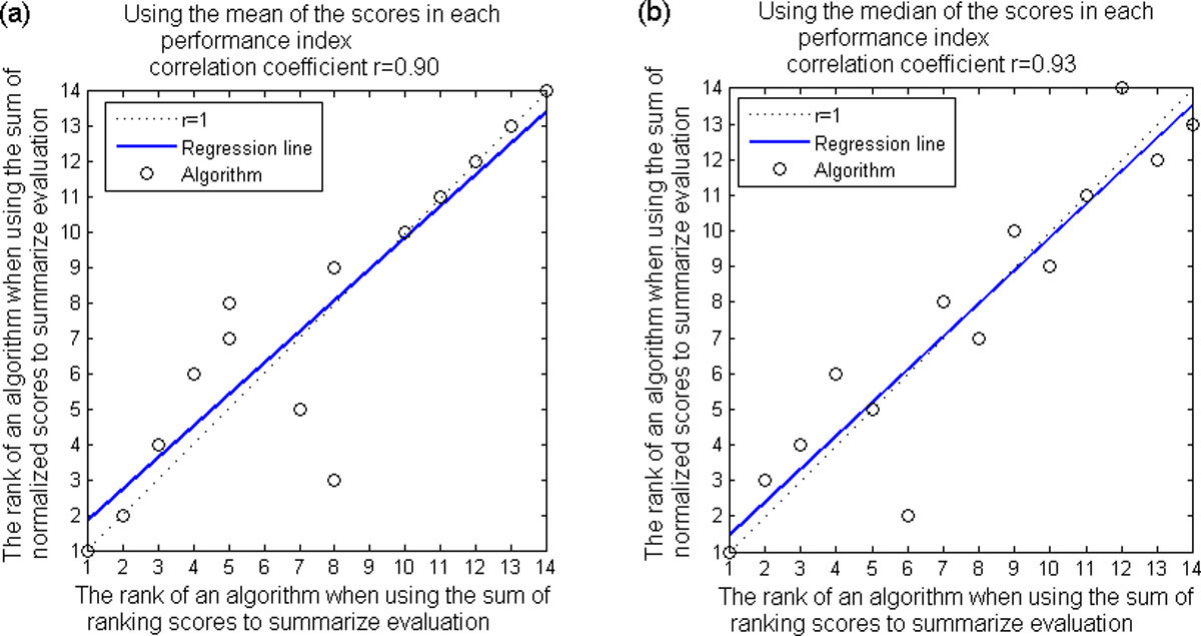
**Robustness against using two different comprehensive ranking measures**. We compared both final ranking lists that resulted from using two comprehensive ranking measures (sum of ranking scores and sum of normalized scores), and obtained (a) correlation coefficient equal to 0.90 (P-value = 1.49e-5 for testing the hypothesis of no correlation) when the mean of the scores in each index is used and (b) correlation coefficient equal to 0.93 (P-value = 1.50e-6) when the median of the scores in each index is used. These results suggest that the comprehensive performance comparison results are robust against using the sum of ranking scores or the sum of normalized scores to summarize evaluation.

Since we implemented two comprehensive ranking measures, sum of ranking scores and sum of normalized scores, for comprehensive performance evaluation on 14 algorithms, we would like to see how the comprehensive performance comparison results change when different comprehensive ranking measures are used. We compared both final ranking lists that resulted from using these two comprehensive ranking measures, and obtained correlation coefficient equal to 0.90 (P-value = 1.49e-5 for testing the hypothesis of no correlation) when the mean of the scores in each index is used. The correlation coefficient is 0.93 (P-value = 1.50e-6) when the median of the scores in each index is used. The linear regression plots are shown in Figure 4a and Figure 4b. These results suggest that the comprehensive performance comparison results are robust against using the sum of ranking scores or the sum of normalized scores to summarize evaluation.

## Conclusions

In this study, we adopted/proposed eight performance indices, of which four are TF-based and four are TG-based, to evaluate and compare the prediction performance of 14 sets of cooperative TF pairs predicted with distinct algorithms. With the comprehensive ranking score or the summarized score assigned to each algorithm, we obtained an objective performance view on the prediction results of the existing cooperative transcription factors identification algorithms. Furthermore, the proposed performance indices can be used as a framework to apply to the predicted cooperative TF pairs in the future study to quickly come out performance evaluation and comparison with previous studies.

## Competing interests

The authors declare that they have no competing interests.

## Authors' contributions

WSW conceived the research topic and provided essential guidance. WSW and FJL developed the method and wrote the manuscript. FJL and HTC did all the simulations. YMH provided advices on the manuscript writing. All authors read, edited and approved the final manuscript.

## Supplementary Material

Additional file 1 **Additional file 1**.xls. This additional file provides the collected sets of predicted cooperative TF pairs (PCTFPs) from 14 existing algorithms in the literature. Click here for file

Additional file 2 **Additional file 2**.xls. This additional file provides the score of each TF pair of the set of PCTFPs under evaluation calculated using eight performance indices. Click here for file

## Declarations

The publication of this manuscript was funded by the Aim for the Top University Project to the National Cheng Kung University.

This article has been published as part of *BMC Systems Biology *Volume 8 Supplement 4, 2014: Thirteenth International Conference on Bioinformatics (InCoB2014): Systems Biology. The full contents of the supplement are available online at http://www.biomedcentral.com/bmcsystbiol/supplements/8/S4.
